# Multifaceted Protective Effects of Hesperidin by Aromatic Hydrocarbon Receptor in Endothelial Cell Injury Induced by Benzo[a]Pyrene

**DOI:** 10.3390/nu14030574

**Published:** 2022-01-28

**Authors:** Juanjuan Duan, Chao Chen, Hong Li, Gaoyan Ju, Ai Gao, Yinghao Sun, Wensheng Zhang

**Affiliations:** 1 Engineering Research Center of Natural Medicine, Ministry of Education, Beijing Normal University at Zhuhai, Zhuhai 519087, China; 306duanjuan@163.com (J.D.); chenchao@bnuz.edu.cn (C.C.); lihongmn@126.com (H.L.); 201921051208@mail.bnu.edu.cn (G.J.); 201921051207@mail.bnu.edu.cn (A.G.); 201920151210@mail.bnu.edu.cn (Y.S.); 2 Zhuhai Branch of State Key Laboratory of Earth Surface Processes and Resource Ecology, Beijing Normal University at Zhuhai, Zhuhai 519087, China; 3 Beijing Key Laboratory of Traditional Chinese Medicine Protection and Utilization, Faculty of Geographical Science, Beijing Normal University, Beijing 100875, China

**Keywords:** hesperidin, benzo[a]pyrene, low-density lipoprotein, atherosclerosis, aromatic hydrocarbon receptor

## Abstract

Benzo[a]pyrene (BaP) causes atherosclerosis by activating the aromatic hydrocarbon receptor (AHR) signaling pathway to trigger lipid peroxidation and inflammation, thereby promoting the development of atherosclerosis. Hesperidin (Hsd), one of the 60 flavonoids of citrus, exhibits therapeutic effects on atherosclerosis. However, its antagonistic function for BaP remains unclear. In this study, the EA.hy926 cell model was used to systematically examine the antagonistic effect of Hsd with BaP, especially in low-density lipoprotein (LDL) oxidation and transport. Results showed that Hsd could reduce BaP-induced AHR activation in mRNA and protein expression level, and reduce LDL accumulation by decreasing the BaP-induced expression of advanced glycation end products and enhancing the BaP-inhibited Adenosine Triphosphate-binding cassette transporter A1 (ABCA1) protein and mRNA expression in EA.hy926 cells. In addition, Hsd could antagonize BaP-induced interaction of reactive oxygen species and the subsequent generation of oxidized LDL and malondialdehyde. Finally, Hsd could alleviate BaP-induced inflammatory response by decreasing IL-1β and TNF-α expression. All these results suggest that Hsd suppresses LDL accumulation, oxidation, and inflammatory response, and thus strongly impedes the AHR pathway activated by BaP.

## 1. Introduction

Atherosclerosis is the primary cause of heart disease and stroke [1], and it begins with certain risk factors, including low-density lipoprotein (LDL) and inflammation. LDL in the bloodstream infiltrates the vascular wall and interacts with reactive oxygen species (ROS) or proteoglycan matrix, thereby causing the retention and modification of LDL to a toxic level in endothelial and subendothelial cells; for example, oxidized low-density lipoprotein (OXLDL) is generated by ROS and results in endothelial cell activation and arterial inflammation [2]. Adenosine Triphosphate-binding cassette transporter A1 (ABCA1) acts as an important membrane transporter that promotes the transfer of cholesterol to apolipoprotein A-I (ApoA-I), which directly contributes to high-density lipoprotein (HDL) production and cholesterol reversal transport [3]. This sequence represents a relevant anti-atherogenic pathway [4]. However, advanced glycation end products (AGEs) inhibit the function of ABCA1, scavenger receptor class B type I (SR-BI) and consequently the cholesterol reversal transport [5,6,7,8]. Therefore, LDL retention and oxidation play a pluripotent and essential role in atherosclerosis.

Numerous epidemiological studies reported that BaP exposure including dietary and inhalation exposure in humans [9] is highly associated with the progression of atherosclerosis [10]. BaP binding to the aromatic hydrocarbon receptor (AHR) activates the AHR pathway, triggers the expression of cytochrome P450 enzymes, such as cytochrome P450 1A1 (CYP1A1), and activates BaP oxidization. 6-OH BaP, one of the intermediate products, is autoxidized to form quinone during ROS production [11,12]. ROS allows the easy transition from LDL to OXLDL to generate reactive carbonyl species (RCS), such as malondialdehyde (MDA). These generated RCS can be further converted into AGEs [13] that stimulate inflammatory responses [14]. Inflammation promotes AGEs production through the myeloperoxidase pathway [15], and AGEs increase ROS production [16]. Some proinflammatory genes containing xenobiotic response elements in the promoter region are directly regulated by AHR, such as interleukin-1β (IL-1β) [17]. AHR also mediates inflammatory signals through nonclassical pathways, such as tumor necrosis factor-α (TNF-α) [18]. Similar to BaP, LDL is a weak activator of AHR [19]. As the first line of defense between circulating environmental toxins and the vascular wall, vascular endothelial cells are susceptible to damage from AHR agonists [20]. Studying the AHR pathway could provide additional molecular biological evidence on the mechanism of BaP-induced atherosclerosis, especially during LDL deposition and oxidation.

Hesperidin (Hsd) is derived from citrus and is a typical bioflavonoid [21] with antioxidant and anti-inflammatory effects [22]. As atherosclerosis treatment, Hsd ameliorates blood lipid profile, inhibits the formation of macrophage foam cells, and alleviates insulin resistance [23] against inflammation and oxidative stress [24]. Moreover, this bioflavonoid attenuates BaP-induced testicular toxicity in rats [25] and affects AHR nuclear translocation [26]. Whether Hsd antagonizes BaP and LDL in endothelial cells, or whether this antagonistic effect involves the AHR pathway remains unclear.

In this study, the multifaced antagonistic effect of Hsd on BaP and LDL was systematically investigated in EA.hy926 cells. Results showed that Hsd exhibits its antagonistic effect by inhibiting LDL accumulation and reducing the oxidation of LDL to OXLDL in endothelial cells, and ROS elimination plays an important role in this process. Given that ROS is mainly produced by BaP metabolism, the regulation of AHR and the subsequent oxidative and inflammatory responses might be the main pathway of Hsd to antagonize cell damage induced by BaP.

## 2. Materials and Methods

### 2.1. Cell Line and Cell Culture

Human EA.hy926 cells were obtained from Cell Resource Center (Peking Union Cell Resource Center, Beijing, China). High-glucose DMEM (Thermo Fisher Co., Waltham, MA, USA) supplemented with 10% fetal bovine serum (Thermo Fisher Co., Waltham, MA, USA), 100 U/mL penicillin, and 100 μg/mL streptomycin (Thermo Fisher Co., Waltham, MA, USA) was used for EA.hy926 culture. The cells were grown in a 37 °C, 5% CO_2_ saturated humidity incubator (Thermo Fisher Co., Waltham, MA, USA) and subcultured with 0.25% trypsin–EDTA (Thermo Fisher Co., Waltham, MA, USA). Cells in the logarithmic growth phase were selected for subsequent experiments.

### 2.2. FITC-LDL Uptake

In brief, 20 μL of FITC (6 mg/mL) and 1 mL of LDL (2 mg/mL) (Yiyuan Biotech Co., Guangzhou, China) were mixed in EP tubes and then incubated at 37 °C for 2 h before being transferred into pre-treated dialysis bags. These bags were dialyzed in PBS for 24 h at 4 °C and protected from light. PBS was changed every other day for three times. Finally, the dialyzed FITC-LDL was transferred into brown EP tubes and stored in a refrigerator at 4 °C before use.

For the cell treatment of LDL uptake, 50 μg/mL FITC-LDL was incorporated with BaP (2.5 μM, B1760, SIGMA, St Louis, MO, USA), methyl β-cyclodextrin (3 mM), aminoguanidine (10 mM), or Hsd (25 μM,50 μM,100 μM, PHR1794, SIGMA, St Louis, MO, USA) as treatment agents. FITC-LDL alone was used as the control. The cells were treated at 37 °C for 3 h. After incubation, the cells were seeded on glass coverslips, incubated at 37 °C for another 3 h, and fixed with 4% paraformaldehyde at room temperature for 15 min). An anti-fluorescence quencher was added to seal the slides (China Beyene Institute of Biotechnology, Nanjing, China). Finally, FITC-LDL uptake was measured by confocal laser scanning microscopy.

### 2.3. Immunocytochemical/Immunofluorescence Imaging

**Figure 1:**
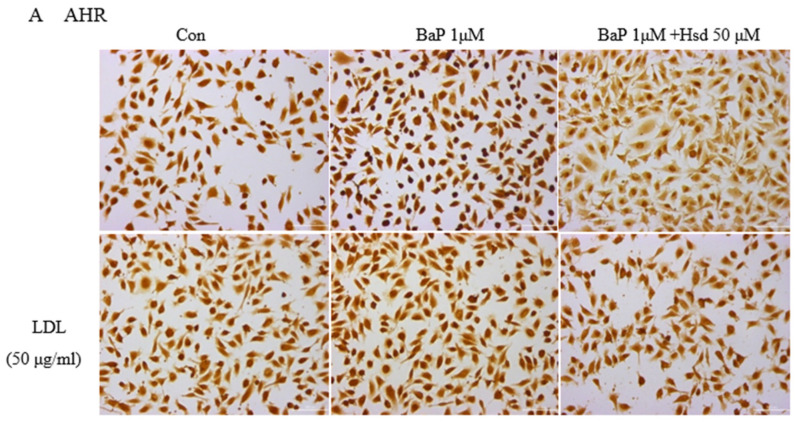

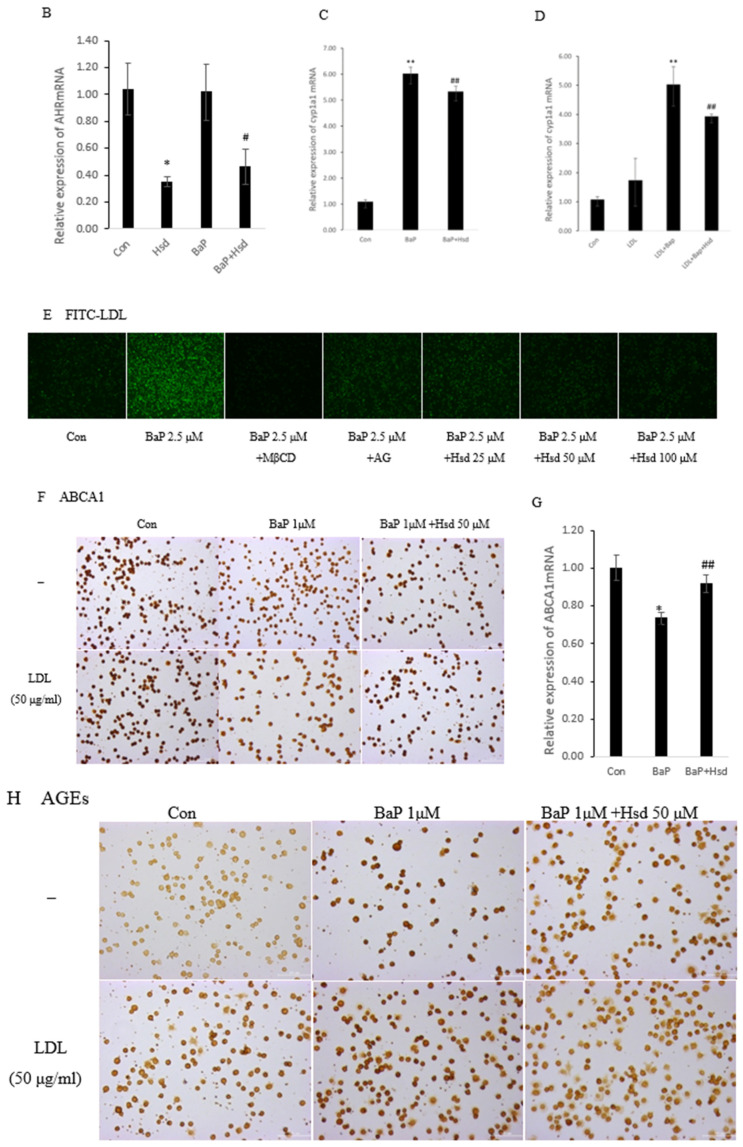
Effect of Hesperidin in mediating low density lipoprotein (LDL) accumulation in EA.hy926 induced by benzo[a]pyrene (BaP). (**A**) Representative immunocytochemistry of aromatic hydrocarbon receptor (AHR) level in EA.hy926 (*n* = 3 per group, Scale bars, 100 μm). (**B**) mRNA levels of AHR in EA.hy926 (*n* = 3 per group). * *p* < 0.05, vs. normal control group, # *p* < 0.05, vs. BaP control group. (**C**) mRNA levels of cytochrome P450 1A1 (CYP1A1) in EA.hy926 (*n* = 3 per group). ** *p* < 0.01 vs. normal control group, ## *p* < 0.01 vs. BaP control group. (**D**) mRNA levels of CYP1A1 in EA.hy926 (*n* = 3 per group). ** *p* < 0.01 vs. LDL control group, ## *p* < 0.01 vs. LDL + BaP group. (**E**) The uptake of FITC-LDL in EA.hy926 was observed with laser confocal microscopy (*n* = 3 per group). (**F**) Representative immunocytochemistry of Adenosine Triphosphate-binding cassette transporter A1 (ABCA1) level in EA.hy926. (*n* = 3 per group, Scale bars, 100 μm). (**G**) mRNA levels of ABC1A1 in EA.hy926 (*n* = 3 per group) * *p* < 0.05, vs. normal control group, ## *p* < 0.01 vs. BaP control group. (**H**) Representative immunocytochemistry of advanced glycation end products (AGEs) level in EA.hy926. (*n* = 3 per group, Scale bars, 100 μm). The data are presented as means ± SD (*n* = 3) and were analyzed using one-way ANOVA. * *p* < 0.05, ** *p* < 0.01 vs. normal control group, # *p* < 0.05, ## *p* < 0.01 vs. BaP control group.

EA.hy926 cells cultured on 24-well plates were divided into six groups and treated with LDL, BaP, and Hsd for 24 h. If there are more than two substances acting on cells, they are added at the same time. (see Figure 1A for grouping details). The cells were then seeded to glass coverslips and incubated at 37 °C for 4 h. After treatment with 4% paraformaldehyde for 15 min, the cells were fixed with 0.5% Triton-X 100 (PBS buffer) for 20 min and with 3% H_2_O_2_ (PBS buffer) for 15 min and finally blocked with 5% goat serum for 20 min at room temperature.

For immunohistochemistry experiments, the cells were incubated with anti-AHR mouse monoclonal primary antibodies (1:200, SC133088, Santa Cruz, CA, USA), anti-ABCA1 mouse monoclonal primary antibodies (1:200, SC53482, Santa Cruz, CA, USA), anti-AGEs rabbit polyclonal primary antibodies (1:200, bs-1158R, BIOSS, Beijing, China), and anti-TNF-α rabbit polyclonal primary antibodies (1: 200, GTX110520, Gentex, MI, USA) overnight at 4 °C. The cells were then incubated with corresponding goat anti-mouse or goat anti-rabbit horseradish peroxidase streptavidin biotinylated secondary antibodies (Santa Cruz, CA, USA) for 20 min and then stained with diaminobenzidine (DAB kit, ZSGB-BIO, Beijing, China). Staining intensity was measured by integrated optical density with Image-Pro Morphometric System (V 6.0). Protein quantity was calculated from the mean of at least three randomly selected slides per group.

For immunofluorescence experiments, the same method was applied. The cells were fixed in 0.5%Triton-X 100 in PBS for 20 min, blocked with 5% goat serum for 20 min at room temperature, and incubated with IL-1β rabbit polyclonal primary antibodies (1:200, ab9722, Abcam, Cambridge, MA, USA) overnight at 4 °C. Afterward, the cells were incubated with FITC-labeled mouse anti-rabbit secondary antibodies (Santa Cruz, CA, USA) for 20 min, followed by the addition of anti-fluorescence quenching blockers (Beyotime Institute of Biotechnology, Nantong, China). Fluorescence intensity was measured by confocal laser scanning microscopy.

### 2.4. Western Blot

EA.hy926 cells were seeded in six-well plates at a density of 5 × 10^4^/mL, incubated at 37 °C for 24 h in a 5% CO_2_ incubator, and treated with BaP (1 μM), LDL (50 μg/mL), or Hsd (50 μM). The cells were incubated for 24 h under the same conditions, washed with cold PBS, and lysed on ice after the addition of RIPA lysis solution (Beijing Sorabio Technology Co., Ltd., Beijing, China) with phenylmethylsulfonyl fluoride (Beyotime Institute of Biotechnology, Beijing, China). After 30 min, the cells were scraped and collected in centrifuge tubes, centrifuged at 12,000× *g* for 10 min at 4 °C. Protein concentration was determined by BCA assay (Beijing Applygen Technology Co., Ltd., Beijing, China). The normalized samples were then loaded onto 12% SDS-PAGE (Anhui Lejin Biotechnology Co., Ltd., Chuzhou, China) and transferred to a nitrocellulose membrane (295 mA, 1.5 h). After incubation with 5% skimmed milk powder solution for 1.5 h at room temperature, protein was detected using anti-OXLDL rabbit polyclonal primary antibodies (1:800, bs-1698R, BIOSS, Beijing, China) and goat anti-rabbit secondary antibodies. Western hybridization membrane was scanned and analyzed with Odyssey 9120 (LI-COR, Inc., Lincoln, NE, USA) with β-actin as the control.

### 2.5. Real-Time qPCR

EA.hy926 cells were seeded in six-well plates at a density of 5 × 10^4^/mL, incubated at 37 °C for 24 h in a 5% CO_2_ incubator, and treated with BaP (2.5 μM), LDL (50 μg/mL), or Hsd (50 μM). Total RNA was extracted from cells according to the manufacturer’s protocol (Invitrogen, Carlsbad, CA, USA). RNA quality was checked by agarose gel electrophoresis. cDNA synthesis was achieved using Go Script™ Reverse Transcription System (Promega, Madison, WI, USA). The primers used for real-time qPCR are given in Table 1. β-actin was used as the housekeeping gene to normalize the expression of the target gene. According to the instructions of Go Taq^®^ qPCR Master Mix PCR kit (Promega, Madison, WI, USA) for RT-qPCR. The eight-tube strip was placed in Applied 7500 for 40 cycles (1 cycle-95 °C for 2 min, 95 °C for 15 s, and 60 °C for 60 s). PCR amplification was performed using Perfecta SYBR green super mix. Ct values were obtained from the software and then the Livak method or the ΔΔCt method were used to calculate the expression fold change relative to the control.

### 2.6. ROS

**Figure 2:**
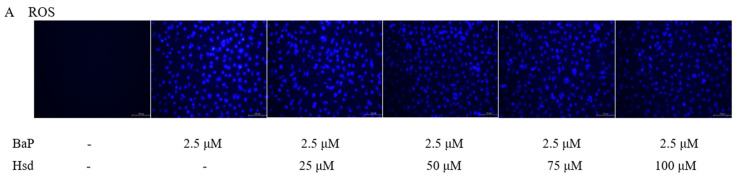

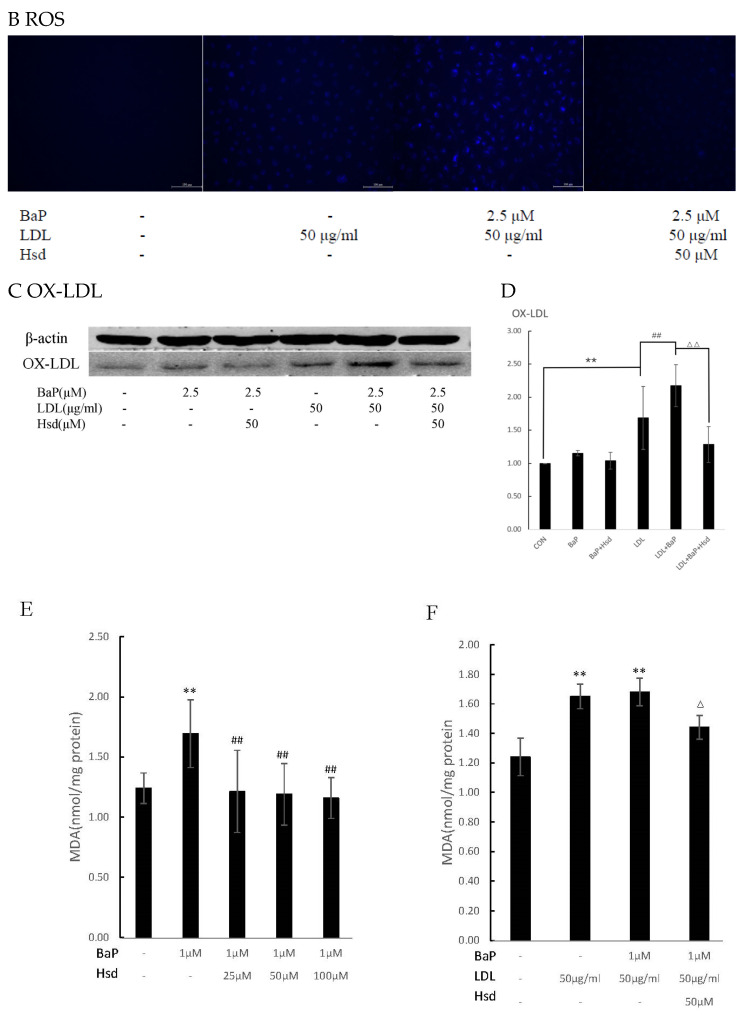
Antioxidant effect of Hesperidin in mediating oxidation in EA.hy926 induced by BaP. (**A**) Effects of Hsd with different concentrations on reactive oxygen species (ROS) production induced by BaP in EA.hy926 (*n* = 3 per group, Scale bars, 100 μm). (**B**) Effect of Hsd on ROS production induced by BaP at high LDL concentrations in EA.hy926 (*n* = 3 per group, Scale bars, 100 μm). (**C**) Representative Western blot of oxidized low-density lipoprotein (OXLDL) levels in the EA.hy926 cells extracts. (**D**) Quantification of OXLDL. The data was presented as means ± SD (*n* = 4), and all experiments were performed in triplicate and analyzed using one-way ANOVA. ** *p* < 0.01 vs. normal control group, ## *p* < 0.01 vs. LDL group, ΔΔ *p* < 0.01 vs. BaP+ LDL group. (**E**) Effect of Hsd with different concentrations on malondialdehyde (MDA) production induced by BaP in EA.hy926. (**F**) Effect of Hsd on MDA production by BaP at high LDL concentrations in EA.hy926. The data are presented as means ± SD (*n* = 3), and were analyzed using one-way ANOVA. ** *p* < 0.01 vs. normal control group, ## *p* < 0.01 vs. BaP control group, Δ *p* < 0.05, ΔΔ *p* < 0.01, vs. BaP+ LDL group.

Intracellular ROS levels were measured in accordance with the instructions of the kit (Nanjing Jiancheng Institute of Biological Engineering, Nanjing, China). The ROS detected included superoxide radicals, hydrogen peroxide, and their downstream products, peroxide and hydroxides After the cells were treated with LDL, BaP, and Hsd for 24 h (see Figure 2A,B for grouping details), the cell medium was removed, and 10 μmol/L DCFH-DA was added. The cells were incubated at 37 °C for 30 min and washed with PBS three times to remove extracellular DCFH-DA. Fluorescence intensity was assessed using a fluorescence microscope (LEICA, Frankfurt, Germany).

### 2.7. MDA

After the cells were treated with LDL, BaP, and Hsd for 24 h (see Figure 2E,F for grouping details), the cell culture medium was removed. The scraped cells were collected into EP tubes, and their MDA content was measured in accordance with the kit instructions (Nanjing Jiancheng Institute of Biological Engineering, Nanjing, China). Total protein concentration was detected by BCA protein quantification kit (Beijing Epson Technology Co., Ltd., Beijing, China).

### 2.8. Statistical Analysis

Data analysis was performed using SPSS version 23.0. Data were presented as means ± standard deviation (SD) from at least three independent experiments repeated three times to obtain the mean. Normally distributed datasets were analyzed with one-way ANOVA, followed by post-Bonferroni’s multiple comparisons test for ≥3 groups. For statistical comparisons, a value of *p* < 0.05 was considered statistically significant and denoted with one- or two-mark symbols when lower than 0.05 or 0.01, respectively.

## 3. Results

### 3.1. Hsd Reduces BaP-Induced AHR Pathway and LDL Accumulation

Given that AHR is the critical receptor for BaP in cells and LDL is a weak activator of AHR, the activation of AHR during LDL accumulation must be analyzed to determine the BaP antagonistic mechanism of Hsd. In Figure 1A, Hsd inhibited the protein expression, of AHR with BaP and LDL. In Figure 1B, Table S1, compared with normal control, Hsd reduced AHRmRNA expression (*p* < 0.05); compared with BaP control, co-treated with Hsd also reduced AHRmRNA expression (*p* < 0.05). In Figure 1C, Table S2, compared with normal control, BaP increased CYP1A1mRNA expression (*p* < 0.01); compared with BaP control, co-treated with Hsd reduced CYP1A1mRNA expression (*p* < 0.01). In Figure 1D, Table S2, compared with LDL control, BaP + LDL increased CYP1A1mRNA expression (*p* < 0.01); compared with BaP + LDL group, co-treated with Hsd reduced CYP1A1mRNA expression (*p* < 0.01). Hsd inhibited the activation of AHR pathway caused by BaP or LDL.

LDL accumulation in vascular endothelial cell is a fundamental cause of atherosclerosis. Analysis was conducted to determine whether BaP alters LDL accumulation in EA.hy926 cells. Compared with that in FITC-LDL control, BaP increased FITC-LDL accumulation in EA.hy926 cells (Figure 1E). By contrast, methylβ-cyclodextrin (MβCD) co-cultured with cholesterol transcellular inhibitors remarkably inhibited BaP-induced LDL accumulation. Consistent with the inhibitory effect of these inhibitors, Hsd also reversed BaP-mediated cellular LDL accumulation in a dose-dependent manner.

In endothelial cells, reverse cholesterol transport can effectively reduce cholesterol accumulation. ABCA1 plays a positive role in this process, whereas AGEs exhibit a suppress effect to some extent [5,6,7,8]. Therefore, changes in ABCA1 and AGEs were analyzed in EA.hy926 cells.

Figure 1F shows that the ABCA1 protein was reduced by BaP treatment in EA.hy926 cells; however, this effect was reversed by Hsd. In addition, compared with the EA.hy926 cells treated with LDL, those treated with a combination of BaP and LDL exhibited decreased ABCA1 expression, and this expression was also reversed by co-cultured with Hsd. Figure 1G, Table S3, shows that ABCA1mRNA was reduced by BaP treatment in EA.hy926 cells; and Hsd increased ABCA1mRNA which was inhibited by BaP. Therefore, Hsd has an antagonistic function against BaP-reduced ABCA1 expression.

Figure 1H shows that BaP and LDL improved AGEs expression in EA.hy926 cells compared with that in normal control. By contrast, Hsd inhibited the BaP-induced increase in AGEs expression. Compared with the BaP group, the cells treated with BaP and aminoguanidine (AG) exhibited suppressed AGEs generation and LDL accumulation (Figure 1E).

### 3.2. Hsd Reduces BaP-Induced Oxidative Activity

ROS is an important byproduct of BaP metabolism through the AHR pathway and an important cause of oxidative stress in cells. As shown in Figure 2A, ROS level was remarkably increased in the EA.hy926 cells treated with BaP but was dose-dependently reduced in the cells treated with Hsd. As shown in Figure 2B, ROS levels were increased by the combination of BaP and LDL but were inhibited by Hsd administration.

ROS expression was lower in the cells with combined BaP and LDL than in those with BaP alone. In the presence of high LDL levels, the majority of intracellular ROS produced by BaP may bind to LDL to form OXLDL, therefore, the less amount of OXLDL in the cells was measured. Figure 2C,D and Figure S1, show that OXLDL levels were significantly increased in EA.hy926 cells treated with BaP alone (*p* < 0.01) or in combination with LDL (*p* < 0.01). By contrast, Hsd inhibited the increase in OXLDL level (*p* < 0.01).

MDA is a byproduct of LDL oxidation that can transform to AGEs. As shown in Figure 2E, Table S4, MDA content in EA.hy926 cells was significantly increased by BaP treatment (*p* < 0.01); however, this effect was dose-dependently inhibited by Hsd administration (*p* < 0.01). In addition, MDA content was increased in EA.hy926 cells co-treated with BaP and LDL (*p* < 0.01), but Hsd counteracted this effect (*p* < 0.05, Figure 2F, Table S4).

### 3.3. Hsd Reduces BaP-Induced Inflammation

**Figure 3:**
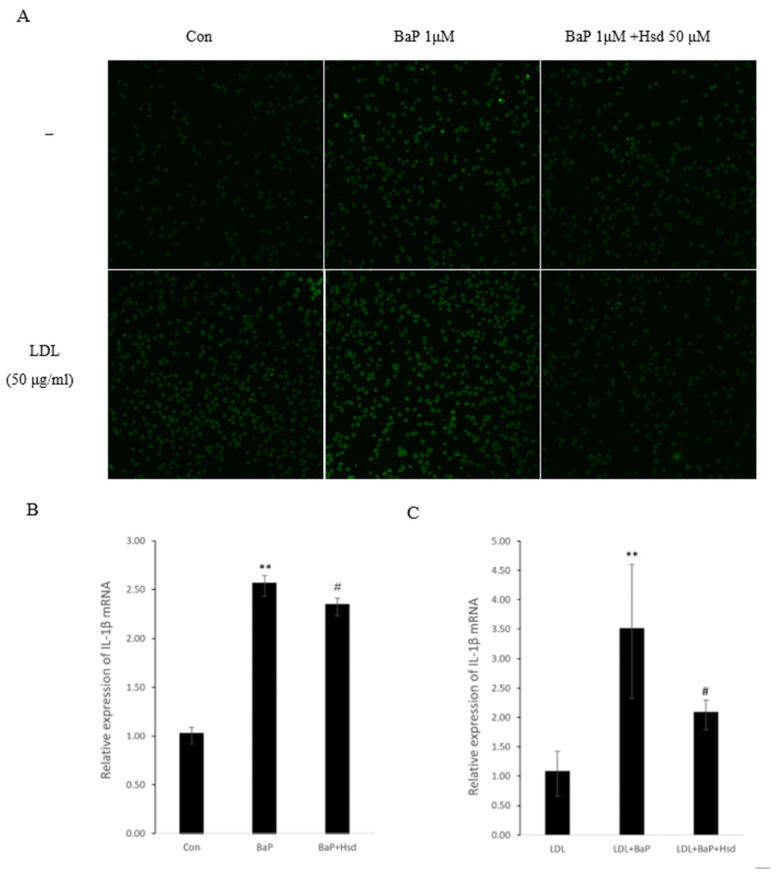

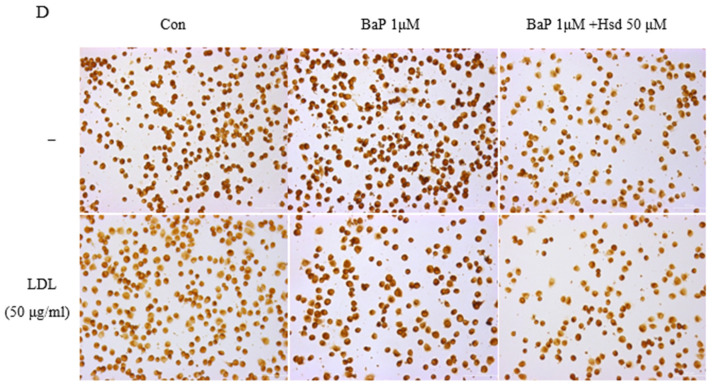
Anti-inflammatory effect of Hesperidin in EA.hy926 induced by BaP. (**A**) Representative Immunofluorescence of interleukin-1β (IL-1β) level in EA.hy926 (*n* = 3 per group). (**B**) mRNA levels of IL-1β in EA.hy926 (*n* = 3 per group). ** *p* < 0.01 vs. normal control group, # *p* < 0.05, vs. BaP control group. (**C**) mRNA levels of IL-1β in EA.hy926(*n* = 3 per group). ** *p* < 0.01 vs.LDL group, # *p* < 0.05, vs. LDL + BaP group. (**D**) Representative immunocytochemistry of TNF-α level in EA.hy926. (*n* = 3 per group, Scale bars, 100 μm). The data are presented as means ± SD (*n* = 3) and were analyzed using one-way ANOVA.

AHR pathway, ROS and AGEs can trigger inflammatory response by producing inflammatory factors such as IL-1β and TNF-α. Inflammation is also an important cause of atherosclerosis. As shown in Figure 3A–C, Table S5, BaP significantly increased the expression level of IL-1β, and Hsd reversed this effect, regardless of the presence of LDL. As shown in Figure 3D, BaP increased TNF-α expression in the presence or absence of LDL, and Hsd suppressed this effect.

## 4. Discussion

Atherosclerosis originates from the accumulation of intimal plaque and cholesterol in the arterial wall. In the initial stages of atherosclerosis, LDL accumulates in the endothelium, which triggers an inflammatory response, and promotes the development of atherosclerosis [27]. ROS converts LDL to reactive OXLDL, making this molecule less recognizable by native LDL receptors [28] and leading to the dysfunction of endothelial, the generation of adhesion molecules, the migration and proliferation of smooth muscle cells, and the formation of foam cells, the hallmarks of the fatty streak phase of atherosclerosis [29]. Dietary exposure to BaP is one of the critical atherogenic pathways [10,30]. With a high-fat diet, the body is at increased risk of being attacked by LDL and BaP in the vascular endothelium, which serves as the first line of defense between circulating environmental toxins and the vascular wall. Studying the response pattern of vascular endothelial cells to BaP and LDL could help uncover the underlying mechanism of atherosclerosis.

In this work, LDL accumulation was significantly increased by BaP treatment (Figure 1E). In LDL cellular uptake, the BaP receptor AHR is activated (Figure 1B–D), and the AGEs levels are elevated (Figure 1H). By contrast, a reduced level was observed for the ABCA1 protein and mRNA, which is responsible for reverse cholesterol transport in vascular endothelial cells (Figure 1F,G). All these cellular responses can be recovered by Hsd treatment. To the author’s knowledge, BaP can bind to AHR as a ligand [31], and LDL is a weak activator of AHR [19]. When AHR is combined with ligand, the expression level of AHR may be slightly decreased [32], but the AHR pathway is activated, for example, the expression level of downstream gene CYP1A1 is significantly increased [33]. AHR is a ligand-activated transcription factor and plays a role in lipid deposition in liver, inflammation, and oxidative processes [31]. In this study, BaP and LDL activate the AHR pathway. In addition to BaP transporting additional LDL into cells, LDL itself aggravates the cell damage caused by BaP metabolic activation. It has been reported that Hesperetin counteracted AHR transactivation and suppressed the downstream gene expression [26]. Meanwhile, the upregulation of CYP1A1 mRNA could be reversed by Hsd, suggesting that its protective effect on BaP- and LDL- mediated endothelial cell injury may be related to the regulation of the AHR pathway.

BaP can generate large amounts of ROS through CYP450 oxidation [11,12], and the excess oxidative capacity generates additional RCS through intracellular LDL oxidation; one example is MDA, which can transform to AGEs by modifying protein [13]. RCS would modify ApoA-I, HDL, and ABCA1, resulting in a significant loss of their ability to support cholesterol efflux [5,7]. Meanwhile, AGEs-albumin could accelerate ABCA1 degradation through proteasome and lysosomal systems [8]. AGEs proteins act as ligands for SR-BI and effectively inhibit the SR-BI-mediated selective uptake of HDL-CE and cholesterol flow from peripheral cells to HDL [6]. However, Hsd can reverse this physiological process. This bioflavonoid acts against AGEs formation at multiple levels through its natural antioxidants, its metal chelating activity, and its ability to trap intermediate dicarbonyl compounds [34]. In addition, Hsd increases ABCA1 expression by activating LXRα and PPARγ [3]. Consistent with previous reports, Hsd enhanced the reverse cholesterol transport that was inhibited by BaP. This regulatory mechanism may be related to the control of additional AGEs production and may also involve the direct effect of Hsd on ABCA1 expression of mRNA and protein.

BaP could be metabolically activated by binding to AHR in the cytoplasm, thereby producing large amount of ROS [35]. AGEs-albumin also increases ROS production by promoting the activities of the NADPH oxidase and mitochondrial system [16]. Excess ROS initiates an inflammatory response, activates NF-κB signaling, and increases chemokine secretion, such as IL-1β and TNF-α [36]. ROS can bind to LDL to form OXLDL and MDA. OXLDL has antigenic potential, contributes heavily to atherosclerosis-associated inflammation [37], and promotes the formation of atherosclerotic plaques [38]. OXLDL cellular accumulation increases oxidative stress, leading to ROS formation. Hsd can neutralizes cellular oxidative stress and enhance cellular antioxidant defense by inducing heme oxygenase-1 (HO-1) and increasing the levels of antioxidant enzymes such as CAT, SOD, and GST through ERK/Nrf2 signaling [22]. In this work, Hsd exhibited anti-ROS activity (Figure 2A,B), reduced OXLDL (Figure 2C,D), and inhibited MDA (Figure 2E,F) formation in BaP-induced cells. Thus, the decreased accumulation of LDL and ROS by Hsd can reduce OXLDL production and subsequently MDA content. Given that ROS partially originates from the regulation of P450 genes by AHR, the role of Hsd in OXLDL regulation may involve the AHR pathway.

OXLDL production is accompanied by a large amount of AGEs. In addition to suppressing the reverse cholesterol transport, AGEs in the cell bind to their receptor, RAGE, which then activates NF-κB into the nucleus and regulates the expression of important target genes, such as IL-1β and TNF-α [39]. Inflammation also increases AGEs expression through the myeloperoxidase pathway [15]. The activation of the AHR pathway and production of ROS in the BaP treated endothelial cells, will lead to an inflammatory response. Hsd shows anti-inflammatory effects under various conditions [40]. In this work, Hsd exhibited its anti-inflammatory activity by decreasing the expression levels of IL-1β (Figure 3A–C) and TNF-α (Figure 3D) in BaP-induced cells.

In this research, ROS acted as a mediator with direct toxic effects and triggered a series of injury responses on cells. This function might have originated from the interference of BaP with the AHR pathway. The activated AHR signaling pathway can cause cellular oxidation, inflammation [29,41], and MDA and AGEs upregulation, which leads to LDL and OXLDL accumulation in endothelial cells. Given that Hsd inhibits AHR expression, its antagonistic effect on BaP-mediated endothelial cell injury is related to the AHR pathway.

## 5. Conclusions

**Figure 4:**
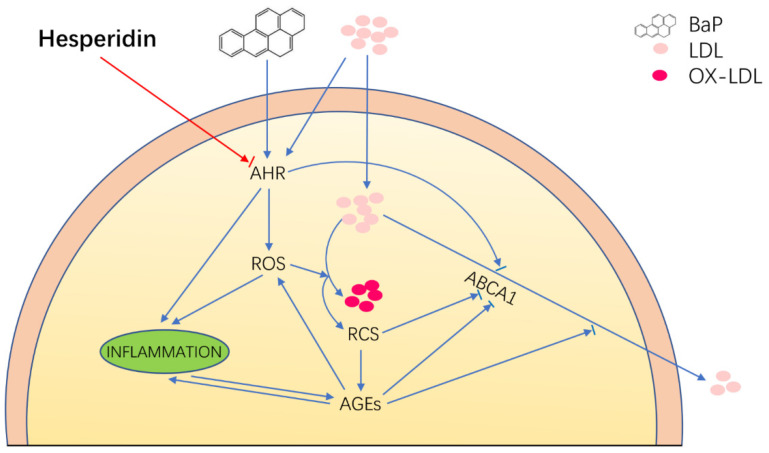
Proposed schematic representation of Hesperidin’s multifaceted protective effects in EA.hy926 cell injury induced by BaP depending on the AHR pathway. The blue indicator line represents the injury response caused by BaP and LDL to EA.hy926 cells, and the red indicator line represents the therapeutic effect of Hsd on EA.hy926 cells injury caused by BAP and LDL. The arrow shows an increase to this indicator’s expression, and the short line before the arrow shows inhibition. Hesperidin attenuated the endothelial cell injury induced by BaP, depending on the AHR-mediated pathway through anti-inflammatory, antioxidant, and enhancing reverse cholesterol transport.

This study reveals the multifaceted protective effects of Hsd on endothelial cell injury induced by BaP and LDL(Figure 4). First, Hsd inhibited AHR activation and reduced LDL accumulation in EA.hy926 cells treated by BaP. Second, Hsd reduced AGEs expression and increased ABCA1 content in the cells, thereby restoring the BaP-inhibited cholesterol reversal transport to reduce LDL accumulation in EA.hy926 cells. Third, Hsd alleviated oxidative stress by reducing BaP-inhibited ROS production, LDL oxidation, and MDA formation. Finally, Hsd decreased the BaP-induced expression of inflammatory factors IL-1β and TNF-α. BaP causes the above damage by activating the AHR pathway, and LDL displays weak AHR agonist properties. Hsd can inhibit the expression of AHR in genome and protein expression level and reduce downstream genes CYP1A1 expression. Moreover, Hsd could reduce the production of ROS, an important product of BaP in AHR metabolism, and also alleviate a series of cell injury caused by ROS. This finding suggests that the AHR pathway plays an essential role in the pathogenesis of Hsd treatment for atherosclerosis caused by BaP and LDL. In summary, Hsd attenuated the endothelial cell injury induced by BaP depending on the AHR by reducing LDL and OXLDL accumulation, enhancing reverse cholesterol transport, and exhibiting antioxidant and anti-inflammatory activities.

## Figures and Tables

**Table 1:** Primers for real-time qPCR.

Name	Sequence (5′-3′)
β-actin-forward	ATCATGTTTGAGACCTTCAACA
β-actin-reverse	CATCTCTTGCTCGAAGTCCA
AHR-forward	CAAATCCTTCCAAGCGGCATA
AHR-reverse	CGCTGAGCCTAAGAACTGAAAG
CYP1A1-forward	TCGGCCACGGAGTTTCTTC
CYP1A1-reverse	GGTCAGCATGTGCCCAATCA
ABCA1-forward	TTCCCGCATTATCTGGAAAGC
ABCA1-reverse	CAAGGTCCATTTCTTGGCTGT
IL-1β-forward	AGCTACGAATCTCCGACCAC
IL-1β-reverse	CGTTATCCCATGTGTCGAAGAA

## Data Availability

The data presented in this study are available in supplementary material.

## Supplementary Materials

The following supporting information can be downloaded at: https://www.mdpi.com/article/10.3390/nu14030574/s1, Table S1: row date of AHRmRNA (RT-qPCR, Figure 1B), Table S2: row date of CYP1A1mRNA (RT-qPCR, Figure 1C,D), Table S3: row date of ABCA1mRNA (RT-qPCR, Figure 1G). Table S4: row date of MDA (Figure 1E,F). Table S5: row date of IL-1βmRNA (RT-qPCR, Figure 3B,C). Figure S1: Raw images of western blot for OXLDL.
