# Supplementary material for: Multifaceted Protective Effects of Hesperidin by Aromatic Hydrocarbon Receptor in Endothelial Cell Injury Induced by Benzo[a]Pyrene
Source: Nutrients. 2022 Jan 28;14(3):574. doi: 10.3390/nu14030574 (PMC8838654; doi:10.3390/nu14030574)
Supplement: Supplementary file 1 [file nutrients-14-00574-s001.zip › nutrients-1524156-supplementary.pdf]

Table S1.row date of AHR mRNA (RT-qPCR)

AHR	△CT	△T	△△T
Con1	8.814333	-0.16189	1.118749
Con2	8.573333	-0.40289	1.322151
Con3	9.541	0.56478	0.676059
BaP1	9.694	0.71778	0.608032
BaP2	8.764	-0.21222	1.158469
BaP3	8.752167	-0.22405	1.168011
BH1	9.437333	0.461113	0.726425
BH2	10.42133	1.445113	0.367263
BH3	10.74567	1.769447	0.293321
Hsd1	10.22067	1.244447	0.42207
Hsd2	10.67033	1.694113	0.309045
Hsd3	10.66133	1.685113	0.310978

Table S2.row date of CYP1A1mRNA (RT-qPCR)

	△CT	△T	△△T
Con1	13.06575	0.21177	0.863477
BaP1	10.28911	-2.56487	5.917006
BaP+Hsd1	10.46419	-2.38979	5.240821
LDL1	11.54108	-1.37957	2.601908
LDL+BaP1	10.61512	-2.30553	4.943505
LDL+BaP+Hsd1	10.8397	-2.01428	4.039779
Con2	12.62208	-0.2319	1.174379
BaP2	10.20226	-2.65172	6.284181
BaP+Hsd2	10.38389	-2.47009	5.540774
LDL2	12.73099	-0.12299	1.088993
LDL+BaP2	10.35865	-2.49533	5.638577
LDL+BaP+Hsd2	10.93187	-1.92211	3.789762
Con3	12.87412	0.020139	0.986137
BaP3	10.35924	-2.49474	5.636271
BaP+Hsd3	10.54094	-2.31304	4.969303
LDL3	12.45065	-0.40333	1.322558
LDL+BaP3	10.75051	-2.10347	4.29742
LDL+BaP+Hsd3	10.94639	-1.90759	3.751824

Table S3.row date ABCA1mRNA(RT-qPCR)

	△CT	△T	△△T
Con1	12.8797	-0.06758	1.033791
Con2	12.90256	-0.04472	1.022359
Con3	13.05957	0.112293	0.943853
BaP1	13.32425	0.376973	0.811513
BaP2	13.41432	0.467038	0.766481
BaP3	13.44798	0.500698	0.749651
BH-1	13.14913	0.201851	0.899075
BH-2	13.06823	0.120952	0.939524
BH-3	12.99632	0.049043	0.975478

Table S4.row date of MDA

name	MDA (nmol/mg protein)
Con-1	1.1000
Con-2	1.2721
Con-3	1.3470
BaP-1	1.8904
BaP-2	1.4190
BaP-3	1.4854
BaP-4	1.9799
BH25-1	0.9223
BH25-2	1.1267
BH25-3	1.5897
BH50-1	0.8987
BH50-2	1.2808
BH50-3	1.3880
BH100-1	1.1376
BH100-2	0.9993
BH100-3	1.3355
LDL1	1.7463
LDL2	1.5888
LDL3	1.6172
BL1	1.5726
BL2	1.7373
BL3	1.7304
BLH-1	1.3756
BLH-2	1.4158
BLH-3	1.5308

Table S5.row date of IL-1β mRNA (RT-qPCR)

AHR	△CT	△T	△△T
Con-1	15.92636	-0.13842	1.1007
BaP-1	14.74344	-1.32134	2.498987
BaP+Hsd-1	14.81192	-1.25286	2.383131
Con-2	16.13302	0.068238	0.953802
BaP-2	14.65426	-1.41052	2.658324
BaP+Hsd-2	14.8241	-1.24068	2.363093
Con-3	16.13498	0.070197	0.952508
BaP-3	14.76694	-1.29784	2.458608
BaP+Hsd-3	14.91609	-1.14869	2.217132
LDL-1	17.81733	0.270439	0.829067
LDL+BaP-1	15.90233	-1.64456	3.126526
LDL+BaP+Hsd-1	16.72675	-0.82014	1.765572
LDL-2	16.97712	-0.56977	1.484287
LDL+BaP-2	15.30374	-2.24315	4.734288
LDL+BaP+Hsd-2	16.36471	-1.18218	2.269187
LDL-3	17.84623	0.299342	0.812623
LDL+BaP-3	16.20126	-1.34563	2.541414
LDL+BaP+Hsd-3	16.48095	-1.06594	2.093537

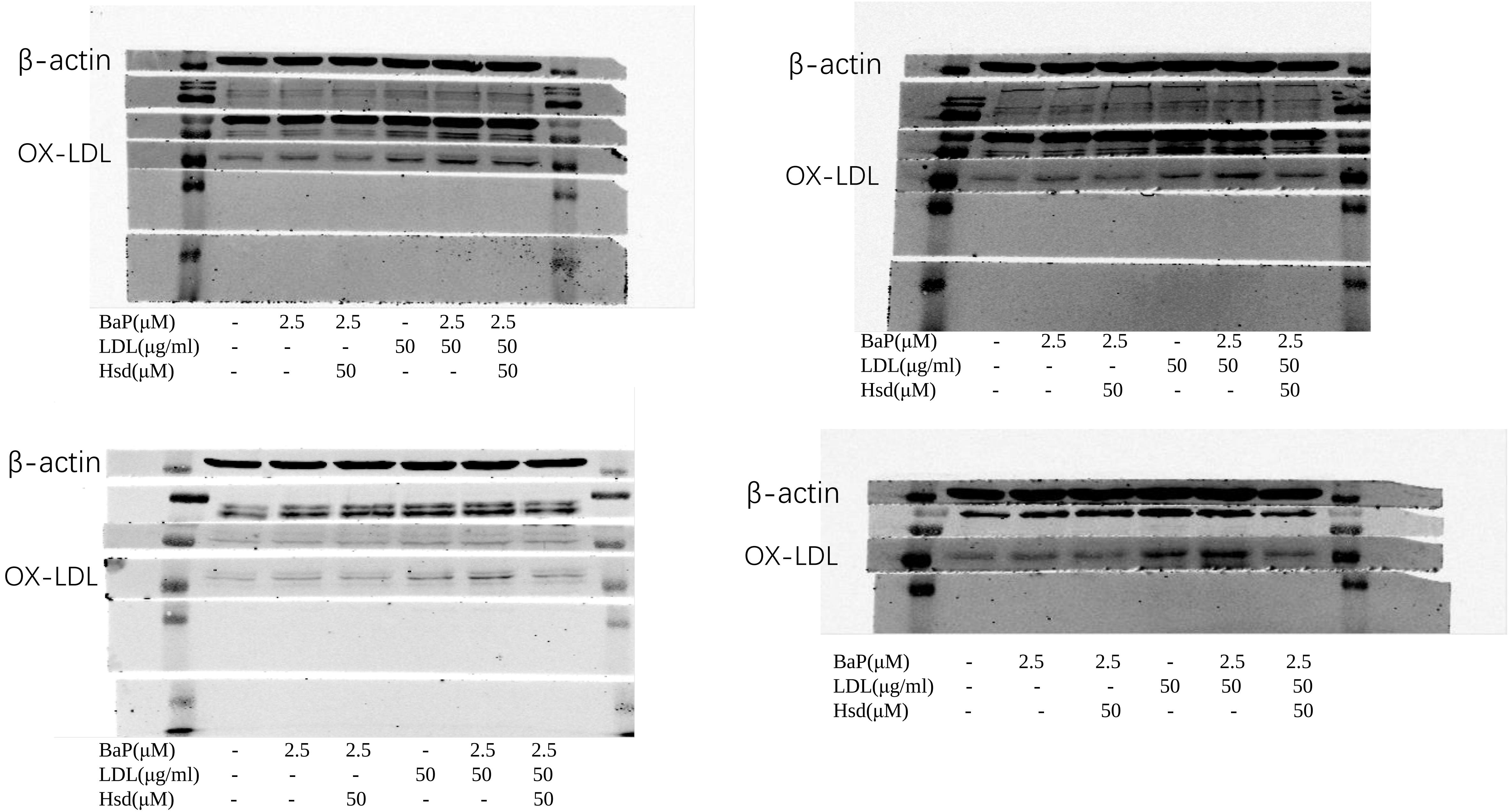

Figure S1 Raw images of western blot for OXLDL
